# A Longitudinal Study of the Impact of Personal and Professional Resources on Nurses’ Work Engagement: A Comparison of Early-Career and Mid-Later-Career Nurses

**DOI:** 10.3390/healthcare11010076

**Published:** 2022-12-27

**Authors:** Satoko Nagai, Yasuko Ogata, Takeshi Yamamoto, Mark Fedyk, Janice F. Bell

**Affiliations:** 1 Department of Gerontological Nursing and Healthcare Systems Management, Graduate School of Health Care Sciences, Tokyo Medical and Dental University (TMDU), Tokyo 113-8510, Japan; 2 School of Health Sciences, Sapporo Medical University, Sapporo 060-8556, Japan; 3 School of Medicine, Betty Irene Moore School of Nursing, University of California, Davis, CA 95817, USA; 4 Betty Irene Moore School of Nursing, University of California, Davis, CA 95817, USA

**Keywords:** antecedent factors, cross-lagged panel design, early-career nurses, personal resources, professional resources, work engagement

## Abstract

To predict and ensure a healthy and high-performing nursing workforce, it is necessary to identify the antecedents that promote work engagement, especially among early-career nurses. To date no study has focused on this. This longitudinal survey, administered to 1204 nurses working in seven general hospitals with 200 or more beds in four prefectures in Japan at two different times in 2019, aims to examine the causal relationship between the personal and professional resources for nurses to work vigorously (PPR-N) and work engagement among nurses in the early stages of their careers, considering time as a key mediating factor. The analysis of structural equation modeling using the cross-lagged effect model supported that PPR-N had significant and positive effects on work engagement after 3 months among early-career nurses with less than 10 years of nursing experience. The PPR-N is a reliable antecedent of work engagement, which is typical of early-career nurses. These results may be provided guidance for managers in overseeing the work environment to ensure a thriving sustainable nursing workforce.

## 1. Introduction

The gap between the supply and demand of nurses is widening owing to emerging infectious diseases and epidemics, and the shortage of nurses remains a global challenge [1,2,3]. A nursing shortage in hospitals reduces the quantity and quality of patient care services. Early-career turnover exacerbates the existing nursing shortages and the cost of recruiting and training new nurses to fill the positions [4].

Nurses—from newcomers to those with 10 years of nursing experience—are generally skilled and critical to patients and organizations as primary providers of nursing care. However, at the start of their careers, nurses must transition through a crucial period [5]. During this period, they are in conflict with their expected roles and the demands of growing as professionals [6]. This critical phase is becoming a problem in the nursing profession. Many nurses’ health deteriorates due to the stress caused by this conflict and anxiety about their future careers, leading them to abandon the nursing profession.

Learning more about work engagement may be the key to solving these challenges. Work engagement is a “work-related positive and fulfilling state of mind, characterized by vigor, dedication, and absorption” [7]. It fosters job retention intention, contributing to staff retention. It also positively impacts nurses’ health and job satisfaction and can enhance their performance [8]. In other words, work engagement promotes workers’ health and organizational performance. Therefore, to ensure a healthy and high-performing nursing workforce, the nurses’ antecedents of work engagement need to be identified, particularly as there are limited findings in previous studies on resources promoting work engagement for early-career nurses.

This study aims to identify the antecedents of work engagement using a comparison of early-career and mid-later-career nurses, considering time as a key mediating factor.

## 2. Background

### 2.1. The Job Demands-Resources Model

According to the Job Demands-Resources Model, job-related and personal resources are linked to positive job outcomes, with work engagement as a mediating factor; it is also an occupational stress model [7]. The Job Demands-Resources Model includes health impairments and motivational processes. The former is referred to as the “health impairment process” and is a process that can cause physical and mental stress reactions at work (job demands) and negative outcomes with respect to health. It is said to be a stressor when one has to work hard to meet the job demands and keep up with expected performance [9]. In the latter, it is referred to as the “motivational process”; job resources and personal resources explain positive outcomes (behaviors) related to organizational commitment and performance through work engagement [9,10]. Based on this model, it can be shown that creating a healthy workplace with high work engagement involves increasing job and personal resources rather than reducing work demands. Therefore, applying this model in this study allows us to examine the relationship between “job and personal resources” and work engagement and to identify the resources that lead to increased work engagement.

The Job Demands-Resources Model has been validated through the analysis of covariance (ANCOVA) using cross-sectional and longitudinal data and has good fit to the data [11].

### 2.2. Work Engagement Outcomes in the Work Environment

Regarding work engagement outcomes in the work environment, we explored the connections between physical and mental health, positive attitudes toward work and one’s organization, and work performance. As for physical and mental health, employees with high work engagement experience less psychological distress and have fewer physical complaints [12]. These outcomes are similar for nurses working in healthcare. The higher the work engagement, the more energy nurses have, and the fewer depressive symptoms they experience [13]. In terms of positive attitudes toward work and one’s organization, the higher the work engagement, the more nurses demonstrate positive expressive behavior at daily meetings and other events [14], and the more they contribute to the workplace outside of work [15].

Regarding work performance, when work engagement is higher, care-related behavior is more patient-centric [16], the quality of care in the department improves [14], patient satisfaction is greater [17], and turnover intention is lower. Thus, it is vital to identify factors that enhance work engagement regarding nurses ‘physical/mental health, turnover prevention, and quality of care.

### 2.3. Work Factors and Individual Antecedents That Promote Work Engagement in Nursing

The determinants of work engagement can be divided into professional and personal resources [9]. Professional resources are found in the workplace; they include managers’ leadership [14], support from superiors and colleagues [11], and the organizational climate. Personal resources include self-efficacy [15], self-esteem [18], autonomous motivation [19], and resilience [20]; they serve to maintain and improve individuals’ work engagement. Previous studies indicate that the more professional and personal resources a worker has, the more their work engagement is enhanced [21]. However, although reports have investigated the causal relationship between resources and work engagement, factors such as “support from superiors and colleagues” may or may not be significant in each survey [22,23]. One reason is that the results of this study may not be stable because of differences in years of experience and nursing team performance. In Knight’s systematic review, to date, about 20 intervention studies have been conducted to improve work engagement. A major challenge is the small effect size of these interventions. A meta-analysis of the effectiveness of interventions to increase work engagement based on 14 controlled studies reported a significant but small overall effect size (Hedges = 0.29, 95%-CI = 0.12–0.46) [24]. One possible reason why these previous studies failed to find large intervention effects on work engagement is that these studies did not employ sufficient strategies to simultaneously improve two important antecedents of work engagement, specifically, job resources and personal resources [9].

Most of the past intervention studies on improving work engagement have focused on programs that focus only on individual resources through cognitive-behavioral approaches [25], and few interventions have focused on job resources. Therefore, research focusing on both job and personal resources is needed. Furthermore, the rankings of the antecedent factors (personal and professional resources) that enhance work engagement differ by occupation [26], reducing the value of extrapolating from other professions to the nursing profession. Hence, it is necessary to identify nurses’ unique antecedents (personal and professional resources) of work engagement that match the nursing profession’s characteristics and the field of practice.

### 2.4. Early-Career Nurses

Previous research has underscored that it is difficult for early-career nurses to settle into the workplace quickly and in a healthy manner [27]. An organization’s quick and easy workplace transition reduces unnecessary turnover, stabilizes human resources, and leads to high-quality patient care [28]. Prior research [20,29] demonstrate that when nurses have higher work engagement, they have lower turnover intentions. It is assumed that even among early career nurses, higher work engagement results in lower turnover rates [20]. However, no studies have examined early-career nurses’ antecedents of work engagement. This seems a critical oversight, as it is imperative to understand how to promote the work engagement of this crucial demographic. Specifically, there is a need to identify the antecedents that enhance nursing professionals’ work engagement. This can be done using a prospective research design to estimate potential causal links [4].

**Figure 1:**
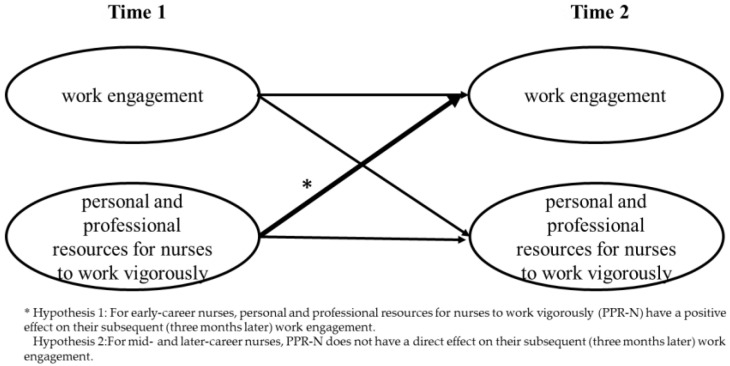
Hypothesized model.

The hypothesis and hypothesized model are presented below. (Figure 1.)

**Hypothesis** **1:** *For early-career nurses, personal and professional resources for nurses to work vigorously (PPR-N) have a positive effect on their subsequent work engagement (three months later).*

**Hypothesis** **2:** *For mid-and later-career nurses, PPR-N does not have a direct effect on their subsequent (three months later) work engagement.*

## 3. Methods

### 3.1. Design

We used a longitudinal research design the core of which is a cross-lagged effect model. Two surveys were administered to the nurses in 2019, and data from these surveys were used to estimate the model.

### 3.2. Participants

**Figure 2:**
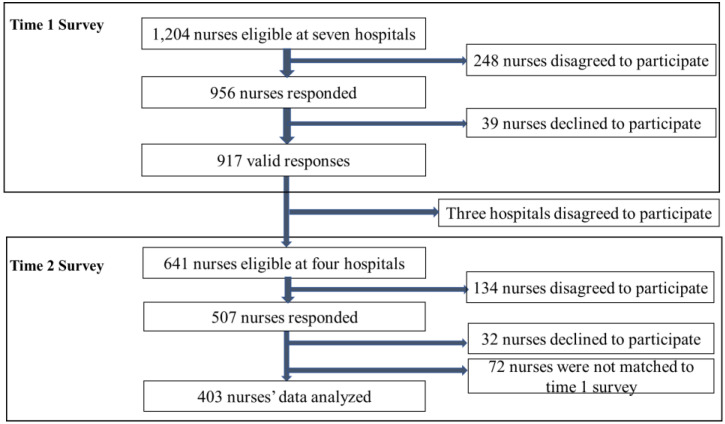
Data collection flow chart.

Using snowball sampling, we selected seven general hospitals with more than 200 beds in four prefectures in Japan. All staff nurses working in the hospitals were surveyed (*n* = 1204). Of the seven hospitals, three were in urban areas, and four were in rural areas. The inclusion criteria were staff nurses with at least 10 months of work experience. Nurse managers, such as division directors, assistant division directors, head nurses, and assistant head nurses were excluded from the study. A total of 956 staff nurses returned the first survey (response rate: 79.4%), and 507 of those who returned the first survey returned the second one (response rate: 53.0%) (see Figure 2).

### 3.3. Data Collection

We collected data using an anonymous questionnaire. In the first survey (February–March 2019), the questionnaires were distributed to nurses by the collaborators (head nurse or deputy) of each hospital, who then collected the sealed questionnaire after completion. The second survey (May–June 2019) was conducted similarly and sent only to nurses who had responded to the first survey. The sealed questionnaires were also collected via the hospital collaborators. Each participant was assigned a unique identifier used on both surveys so that the surveys could be linked without identifying the participant. The cross-lagged design requires that we assess the correlation between variables measured at two different times. To this end, we used PPR-N as a predictor of work engagement among nurses, measured in two different surveys administered three months apart. In the following, Time1 corresponds to measures derived from data collected from February–March 2019, and Time2 corresponds to measures derived from data collected from May–June 2019. In addition, in prior studies, job crafting [29] and a web-based stress and depression literacy [30] interventions were reported to significantly improve work engagement after three or four months. Therefore, we surveyed twice after a time frame of three months later for this study, based on these studies.

### 3.4. Pilot Study

Prior to the pilot study, surface validity was ensured by checking the wording of the questionnaire and the ease of answering. There were six participants: four graduate students majoring in nursing and two nurses working at a hospital. We revised the text and structure of the questions in the questionnaire, as indicated by the participants.

The pilot study was conducted from April to May 2018, and 88 staff nurses returned the questionnaire (collection rate: 32.0%). The data from this survey were used to confirm the reliability and validity of items and to verify the analysis method used in the main survey. 

In addition to the pilot studies, we applied the total design method [31] to improve the mail survey’s response rate by using the cumulative response proportion [32]. 

### 3.5. Instruments

#### 3.5.1. The Utrecht Work Engagement Scale

We used the Japanese version of the Utrecht Work Engagement Scale to measure work engagement [33]. The scale’s items are rated on a 7-point Likert scale ranging from 0 = *never* to 6 = *every day*. The nine work engagement items consist of three subscales: vigor, dedication, and absorption. The score is calculated as the average of all the that comprising each factor. The higher the score, the higher the level of engagement. Confirmatory factor analysis supports the scale’s 3-factor structure. The validity and reliability of the scale have been tested in various occupations and are very useful as they can be employed for inter-occupational and global comparisons [33].

#### 3.5.2. Personal and Professional Resources for Nurses to Work Vigorously (PPR-N)

This study’s main construct of interest—PPR-N—was derived from a content analysis of interviews with ten nurses, from which a set of items was derived [34]. In these interviews, they described their work resources (workplace characteristics) and personal resources (e.g., their position in the workplace) as antecedents to work engagement. 

Four nurses with nursing education experience examined these items to determine whether the terminology was easy to understand, whether the wording was appropriate in actual use, and also whether the wording of the items was refined (surface validity was confirmed). Subsequently, as a pilot test, a questionnaire survey of 275 nursing staff at a medium-sized general hospital in Japan was conducted on the PPR-N items (Medical Research Ethics Committee of Tokyo Medical and Dental University, approval number: M2017-321). The results of the ceiling effect, floor effect, and item-total correlation analysis indicated that none of the items deviated from the reference values. Exploratory factor analysis (EFA) revealed a three-factor structure (KMO = 0.64). The alpha coefficient was 0.89, confirming internal consistency. Through these processes, PPR-N was confirmed to have certain validity.

Specifically, the PPR-N consists of 14 items, each rated on a 7-point Likert scale ranging from 0 = *never* to 6 = *always*. It has three subscales: “work environment with peace of mind” (6 items), “efforts that lead to good outcomes” (5 items), and “prospects for moving forward” (3 items). Its scores are calculated by summing and averaging the item scores for each factor and then calculating the scores for “job resources” and “personal resources” (Appendix A).

#### 3.5.3. Early/Mid-and Later-Career Nurses

In the career development process, Schein [5] considers a career to be one’s work experience throughout life and divided the career cycle into nine stages according to how general or specific the problems an individual typically faces and has the skills or resources to overcome. Early-career individuals share a set of problems common to all the individuals, while later-career individuals face much more specific problems. The process from entry-level to approximately ten years of work experience is the early stage of a career [35], with career development challenges such as “successfully performing one’s job”, “acquiring specialized skills and knowledge to lay the foundation for cross-disciplinary career growth”, and “seeking to develop the skills and knowledge needed to succeed in the workplace” and other career development issues [5]. 

Furthermore, the duration of approximately ten years of work experience, beginning with a new employee, is a critical period in the career development of nurses, as this duration shapes the career orientation of nurses as well as their identities [36,37].

Based on Schein’s (1991) career classification, this study defined nurses in the early stages of their career as “having less than 10 years of experience” [5,35], while nurses in the middle or later stages were defined as “having more than 10 years of experience”. 

### 3.6. Data Analysis

To test whether a model exhibits a cross-lagged effect—and thus whether a causal relationship exists between two or more time-separated variables—we must first construct a structural equation model of the data [38]. We accomplished this with a model employing maximum likelihood estimation. Specifically, we modeled work engagement as a latent variable and used the subfactors (vigor, dedication, and absorption) as reflective indicators. We modeled PPR-N as a latent variable and used the subfactors (“work environment with peace of mind”, “efforts that lead to good outcomes”, and “prospects for moving forward”) as reflective indicators. We set the critical values of the comparative fit index [39] and the Tucker–Lewis index to 0.90. Root mean square error of approximation evaluates the degree to which a model does not fit; if it is smaller than 0.06, the model has good fit [40]. Descriptive statistics were performed using SPSS version 27 (IBM). This structural equation model then allows us to assess whether a cross-lagged effect exists in our data. To do this, we used AMOS 25.0 to analyze our data.

### 3.7. Validity, Reliability, and Rigor of Measurement Scales

The Cronbach’s alpha was 0.935 on work engagement. An exploratory factor analysis of the PPR-N items revealed a 14-item, 3-factor structure with a Cronbach’s alpha of 0.930. Work engagement and PPR-N showed high internal consistency.

### 3.8. Ethical Considerations

This study was approved by the Medical Research Ethics Committee of Tokyo Medical and Dental University (approval number: M2017-321; M2018-215). Requests were made to the person in charge of the nursing department of the relevant hospitals inviting their nurses to participate. They were invited to participate by distributing flyers to all eligible nurses through collaborators at each hospital. We explained to the participants the purpose and method of the study, privacy policy, the voluntary nature of participation, and that they would not be disadvantaged by not participating or withdrawing their consent after completing the survey. Consent was obtained from all study participants. 

## 4. Results

Table 1 outlines the demographic characteristics of the sample according to work experience. Four hundred three staff nurses were included in the study (see Figure 1). On average, respondents were 40.1 years old (SD = 11.9) and had 15.7 years of experience (SD = 10.5). The majority were female (90.6%), with 80.6% graduating from a vocational school or junior college and over 90% working full-time. They worked in various patient care facilities (e.g., acute, specialty, sub-acute, long-term care, etc.) On average, early-career nurses had 5.7 years of work experience (SD = 2.7) and were in their 20 s (61.0%) and 30 s (34.0%). Most early-career nurses had gone to vocational school or junior college (92.2%). The mid-career nurse participants had an average of 22.3 years of work experience (SD = 8.2). Nurses aged in their 40 s (41.8%) were most common in this group.

Table 2 outlines the descriptive statistics for work engagement and personal and professional resources for nurses to work vigorously by years of work experience.

### 4.1. Work Engagement Scores Based on Years of Work Experience at Time 1 and Time 2 

The mean work engagement score of the 403 staff nurses at Time1 was 2.51 (SD = 1.02). The highest scoring work engagement factor was “dedication”, and the lowest was “vigor”. Nurses with a minimum of 10 years of experience had significantly higher total work engagement scores than nurses with less than 10 years of experience (mean: 2.65 and 2.28, respectively, *p* < 0.001). The mean scores for the three work engagement factors were higher for nurses with more experience. The mean scores for the three-work engagement subfactors were higher at Time1 than at Time2 for both the early- and mid-career nurse cohorts. However, the “absorption” factor for early-career staff nurses was lower at Time1 (mean: 2.09 [SD: 1.13]) than at Time2 (mean: 2.13 [SD: 0.83]).

### 4.2. Item Group Scores for Factors Preceding Work Engagement Based on Years of Work Experience at Time 1 and Time 2

The 403 staff nurses had a mean PPR-N score of 4.08 (SD = 0.76) at Time1. The total PPR-N score at Time1 was significantly higher (*p* < 0.001) for early-career staff nurses (mean: 4.09 [SD: 0.81]) than for mid- and later-career staff nurses (mean: 4.07 [SD: 0.73]). The mean score for each PPR-N factor was significantly higher at Time1 than at Time2 for both groups of nurses in the early- and mid-career stages (*p* < 0.001). The mean scores for the PPR-N factors, “efforts leading to good outcomes”, and “prospects in the profession” were higher in the group with more years of work experience, while the mean score for “work environment with peace of mind” was lower in the same group.

### 4.3. Hypothesis Testing: Cross-Lagged Effects Model

**Figure 3:**
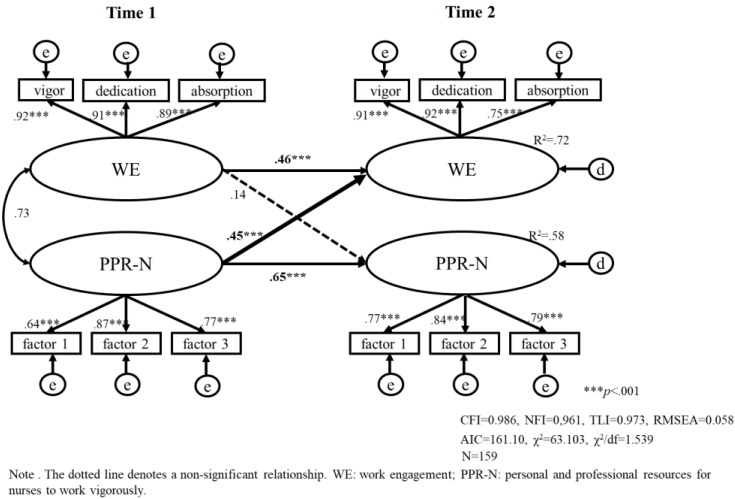
Model (a). The causal relationship between work engagement and personal and professional resources for nurses to work vigorously using a cross-lagged effects model in nurses with less than 10 years of work experience (early-career nurses).

**Figure 4:**
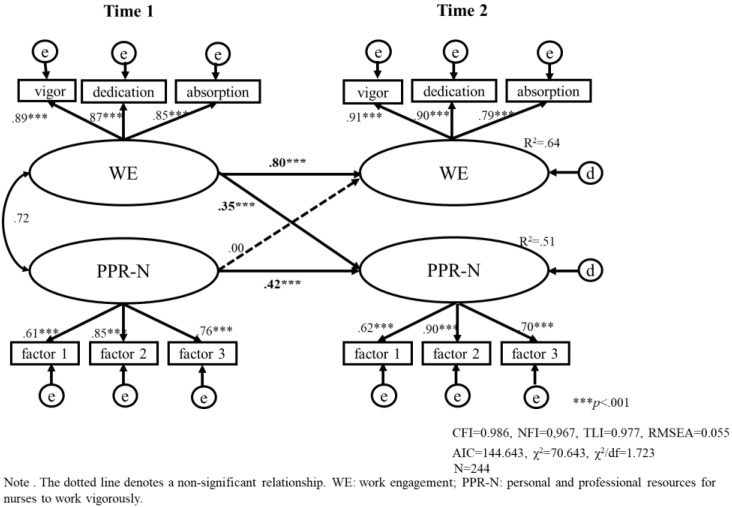
Model (b). The causal relationship between work engagement and personal and professional resources for nurses to work vigorously using a cross-lagged effects model in nurses with more than 10 years of work experience (mid- and later-career nurses).

We tested two models (model a, b) that included a stability effect and a cross-lagged relationship between PPR-N and work engagement in a cohort of early-career nurses and mid-later-career nurses. For early-career nurses, 159 were included, and the results of the analysis are shown in Figure 3 (model a); for mid- to late-career nurses, 244 were included, and the results are shown in Figure 4 (model b). As can be seen in Figure 3 and Figure 4, all estimated structural models fit well (Figure 3: CFI = 0.986, NFI = 0.961, RMSEA = 0.058, Figure 4: CFI = 0.986, NFI = 0.967, RMSEA = 0.055). 

The PPR-N at Time1 was positively correlated with work engagement at Time 2 (β = 0.45, *p* < 0.001) for the early-career nurses. Work engagement at Time1 was independent of PPR-N at Time2 (β = 0.14, ns) for the early-career nurses (see Figure 3). From these we test Hypotheses 1; PPR-N has a positive (cross-lagged) effect on their subsequent (three months later) work engagement, and there is no direct effect of work engagement on PPR-N held for the early-career nurses’ population. In brief, it is clear that PPR-N is an antecedent resource of work engagement, and work engagement is not an antecedent of PPR-N among early-career nurses.

The PPR-N at Time1 was unrelated to work engagement at Time 2 (β = 0.00, ns), and work engagement at Time1 was positively correlated with PPR-N at Time2 for mid- and later-career nurses (β = 0.35, *p* < 0.001) (see Figure 4). From these, we test Hypothesis 2; there is no direct effect of PPR-N on their subsequent (three months later) work engagement for mid- and later-career nurses. In addition, there is direct effect from work engagement to PPR-N for nurses in mid-career and beyond. 

In brief, for early-career nurses, by comparing with mid- and later-career nurses, PPR-N influenced work engagement and was a predictor of work engagement. In contrast, PPR-N did not directly affect work engagement among mid- and later-career nurses. 

## 5. Discussion

The present study is the first to investigate the concept of predicting work engagement among early-career nurses, by comparing nurses in the early-career stage to those in later stages. Our data support the conclusion that for nurses at the beginning of their career, their commitment and dedication (i.e., the strength of their work engagement) will be enhanced by work environments that supply adequate professional resources. 

### 5.1. The Characteristics of Nurses’ Work Engagement

The mean work engagement score of all participants in this study was 2.51 points, lower than the mean score of 3.96 points in the US [41], 4.1 points in Saudi Arabia [42], 3.13 points in China [43], and scores reported in previous studies. The low work engagement in Japan may be related to nurses’ workload rather than a shortage of nurses. In addition, this study took place in Japan, where it is culturally desirable to suppress positive emotions and attitudes [12]. This may have led to a similar tendency in this study, and the work engagement scores may have been lower than for nurses outside Japan.

The highest score among the three subscales was “dedication”, consistent with a national survey of 1194 registered nurses in Japan [44]. Similarly, in a work engagement survey of registered nurses (*n* = 747) providing direct patient care at five rural acute care hospitals in the US, the highest subscale for any generation of nurses was “dedication”. For early-career nurses in the present study, the next highest score on the three subscales after dedication was “absorption”, and the lowest was “vigor”. Previous literature has found that employees with low dedication and vigor have strong turnover intention, especially when the workload is high. Therefore, nurse managers need to be aware of subordinates’ potential intent to leave when the total score of it is relatively low. Vigor is the lowest of the subfactors, as found in the participants of this study.

Participants with more than 10 years of experience had significantly higher work engagement scores than those with less than 10 years of experience, consistent with an earlier study of 1194 registered nurses in Japan [44]. Past studies indicate that work engagement scores rise as years of experience increase. Nurses with more than 10 years of experience may be less likely to feel burdened by their workload due to their proficiency in their roles, which may have increased work engagement.

The mean of the total work engagement scores was higher at Time1 than at Time2. This outcome is similar to the findings of a longitudinal study involving Chinese female nurses with a six-month gap between surveys [45] and a longitudinal study of Canadian nurses with a one year gap between surveys [19]. Possible reasons for this have not been mentioned in prior studies and require further investigation.

### 5.2. Comparison of Resources to Promote Work Engagement for Early-Career and Mid-Later Career Nurses

In this study, the PPR-N was indicated to be effective for work engagement after three months among nurses with less than ten years of experience, and the PPR-N was not found to be effective in work engagement among nurses with more than ten years of experience. This may have been due to the fact that the PPR-N was a group of items obtained through qualitative analysis of interviews with nurses with almost exclusively less than ten years of experience. Furthermore, compared to nurses with less than ten years, nurses with more than ten years are in what Schein describes as the “mid-career crisis” stage [5,36], which involves career challenges such as “reaffirming the position of their career in their overall life” and “accepting reality and deciding to continue working. This may have affected the difference in results between the two groups because the challenges are different in the “early career” stage, which is to “perform tasks successfully” and “remain in the organization or seek harmony between organizational constraints/opportunities and one’s own desires”, compared to the mid-career stage, where the challenges are different. 

Nurses with more than ten years of experience had higher work engagement scores after three months than nurses with less than ten years of experience, and their work engagement remained relatively stable after three months (β = 0.80, *p* < 0.001). In addition, work engagement affects PPR-N among this mid-and later-career nurses. These suggest the possibility that nurses with more than ten years of experience may have a job and personal resources other than PPR-N that promote work engagement. For example, a prior study [44,46] has stated that veteran nurses with ten or more years of experience have a higher degree of confidence in their work due to their greater experience and that these personal resources increase work engagement. 

### 5.3. The Usefulness of Personal and Professional Resources for Nurses to Work Vigorously in the Nursing Field

Although the period between Time1 and Time2 in this study was brief (three months, as noted), it was a time of fluctuation due to nursing staff turnover because it spanned the entire fiscal year. Some staff members and new nurses were not yet accustomed to their work, and the PPR-N may have been implemented during a busy period. Regardless, it was still a leading factor in work engagement. In other words, PPR-N may be a stable antecedent of work engagement not substantially impacted by the degree of activity in the ward.

### 5.4. Relevance for Clinical Practice

Our results suggest that strengthening the support provided for nurses in areas linked to our PPR-N construct could enhance the work engagement of early-career nurses with less than ten years of experience. Enhancing nurses’ work engagement is a complex issue for organizations. However, our data suggest that this complexity can be simplified using a career cycle (time of career) focused approach to enable the deployment of targets and resources [41]. Nurse managers are best positioned to improve the work-life of their subordinates as they are more familiar with the problems and conditions faced in the unit. Therefore, in the future, managers can employ resources that enhance the work engagement of early-career nurses, implement these resources at the appropriate time, and prepare a supportive work environment in which nurses can work vigorously. Ultimately, early-career nurses can continue to work without interruptions in their careers. They can improve their performance, enhance the quality of care provided by the nursing profession, and nurture a better medical and healthcare environment.

### 5.5. Limitations of the Study and Future Research

This study has several limitations. First, the PPR-N was limited only to the nursing work environment in Japan. As such, the roles of nurses in Japan may be different from those of other countries. For future studies, it is necessary to examine to confirm the cultural applicability of the model. Second, this study only considered the point of view on the work engagement effectiveness of PPR-N. To examine the effects of work engagement more comprehensively, future research should consider other antecedent factors as well, such as staff nurses’ performance. Third, the design was longitudinal, and causality was estimated in two intervals. In the future, it will be necessary to change the time period and further design the study at a different time point (Time3) and at a medium- to long-term interval to test the usability of the model. 

## 6. Conclusions

We aimed to determine the antecedents of work engagement using a comparison of early-career and mid-later-career nurses considering, using time as a mediating effect. Our results supported the hypotheses and indicated that in early-career nurses, PPR-N has a significant and positive effect on work engagement. However, PPR-N in mid- and later-career nurses with more than ten years of experience did not promote work engagement. It may also be an antecedent to work engagement among early-career nurses with less than ten years of work experience. Increasing PPR-N resources increases the possibility of promoting promote work engagement. Our results can guide managers in predicting and implementing beneficent work environment measures to enable nurses to work more effectively.

## Figures and Tables

**Table 1:** Demographic traits of the participants according to years of work experience (Time 1).

		Total (*n* = 403)			0–10 Years (*n* = 159)			10 Years and More (*n* = 244)		
Variables	Categories	Mean ± SD	*n*	%	Mean ± SD	*n*	%	Mean ± SD ^†^	*n*	%
Age		40.1 ± 11.9	403		29.5 ± 6.1	159	100.0	46.8 ± 8.7	244	100.0
	20–29		98	24.3		97	61.0		1	0.0
	30–39		110	27.3		54	34.0		56	23.0
	40–49		106	26.3		4	2.5		102	41.8
	50–59		63	15.6		3	1.9		60	24.6
	60-		26	6.5		1	0.6		25	10.2
Sex	Male		38	9.4		24	15.1		14	5.7
	Female		365	90.6		135	84.9		230	94.3
Years of experience in nursing		15.7± 10.5	403		5.7 ± 2.7	159		22.3 ± 8.2	244	
Years of experience at the hospital		12.1 ± 13.4	403		5.9 ± 13.2	159		14.7 ± 12.3	244	
Education	Vocational school or junior college		325	80.6		100	63.7		225	92.2
	Undergraduate or graduate school		73	18.1		57	36.3		16	6.6

^†^ SD: Standard deviation. 0–10 years: *n* = 159, 10 years and more: *n* = 244.

**Table 2:** Descriptive statistics for work engagement and personal and professional resources for nurses to work vigorously by years of work experience.

Years of Work Experience	All (*n* = 403)		0–10 Years (*n* = 159)		10 Years and More (*n* = 244)	
	Time 1	Time 2	Time 1	Time 2	Time 1	Time 2
Variables	Mean ± SD ^†^	Mean ± SD	Mean ± SD	Mean ± SD	Mean ± SD	Mean ± SD
UWES ^‡^ [range: 0–6]	2.51 ± 1.02	2.44 ± 0.88	2.28 ± 0.99	2.24 ± 0.88	2.65 ± 1.01	2.57 ± 0.86
Vigor [range: 0–6]	2.25 ± 1.10	2.21 ± 1.05	2.04 ± 1.03	1.96 ± 1.01	2.39 ± 1.12	2.37 ± 1.04
Dedication [range: 0–6]	2.97 ± 1.04	2.86 ± 1.01	2.72 ± 1.01	2.64 ± 1.05	3.13 ± 1.03	3.00 ± 0.95
Absorption [range: 0–6]	2.30 ± 1.17	2.26 ± 0.83	2.09 ± 1.13	2.13 ± 0.83	2.44 ± 1.17	2.34 ± 0.83
^§^ PPR-N [range: 0–6]	4.08 ± 0.76	3.97 ± 0.75	4.09 ± 0.81	3.94 ± 0.80	4.07 ± 0.73	3.98 ± 0.71
Work environment with peace of mind [range: 0–6]	4.32 ± 1.01	4.18 ± 0.94	4.38 ± 1.05	4.23 ± 0.99	4.27 ± 0.98	4.15 ± 0.91
Efforts that lead to good outcomes [range: 0–6]	3.73 ± 0.84	3.64 ±0.86	3.70 ± 0.92	3.59 ± 0.92	3.75 ± 0.78	3.67 ± 0.82
Prospects for moving forward [range: 0–6]	4.19 ± 0.76	4.08 ± 0.74	4.15 ± 0.75	3.95 ± 0.73	4.22 ± 0.76	4.16 ± 0.74

^†^ SD: Standard deviation, ^‡^ UWES: Utrecht Work Engagement Scale, ^§^ PPR-N: personal and professional resources for nurses to work vigorously.

## Data Availability

Data sharing is not applicable to this article because we have not obtained approval of the participants or the Ethics Committee to release the data.

## Appendix A

Personal and professional resources for nurses to work vigorously

The following 14 statements describe how you may feel about your job: read each statement carefully, and rate how you feel about each statement. Choose the option that best describes your experience at your work. If you cannot relate to the statement, please circle the number 0, if you relate to the statement, please circle the number from 0 to 6 depending on how often.

Items of personal and professional resources for nurses to work vigorously

Factor 1: Work environment with peace of mind (6 items)
I am surrounded by coworkers who will support me in times of difficulty.
There are positive relationships within the workplace.
The workplace environment is accepting and tolerant toward anybody to speak up.
I feel comfortable talking to other staff members.
I feel a sense of belonging at my workplace.
There is a positive atmosphere at the workplace.
Factor 2: Efforts that lead to good outcomes (5 items)
There are jobs that stimulate my interest.
I receive feedback on the accomplishments that I have worked for.
My efforts lead to good outcomes.
I can use my strengths and abilities to assist others.
I feel that my efforts and works are going well.
Factor 3: Prospects for moving forward (3 items)
I am able to predict what I need to do next based on what I have learned in the past.
I am making progress towards becoming the nurse that I strive to be in the future.
I have a sense of ownership in the workplace.
Note. Choices 0: never, 1: rarely, 2: occasionally, 3: sometimes, 4: often, 5: usually, 6: always.
